# Dependence of scar contrast in LGE images on the time interval after contrast injection

**DOI:** 10.1186/1532-429X-13-S1-P42

**Published:** 2011-02-02

**Authors:** Sathya Vijayakumar, Eugene G Kholmovski, Nassir F Marrouche

**Affiliations:** 1 University of Utah, Salt Lake City, UT, USA

## Aim

To study the dependence of post-ablation scar visibility in late gadolinium enhancement (LGE) images of left arial wall on time interval after contrast agent injection

## Introduction

It was shown that LGE imaging can be used to evaluate post-ablation scar [1,2] and pre-ablation remodeling of left atrium [3]. Visibility of scar depends on a time interval between contrast agent injection and LGE scan. In this study, we try to determine, what time post contrast injection would give optimal contrast between post-ablation scar and blood (CNR_SB_) and scar and normal myocardium (CNR_SM_).

## Methods

The study was performed retrospectively on 3-month post ablation LGE data acquired on a 3T Verio scanner (Siemens Healthcare, Erlangen, Germany) with a full dose (0.1mmol/kg) of contrast agent (Multihance, Bracco Diagnostic Inc., Princeton, NJ). The data were separated into 3 groups based on time post contrast:

1) 20-30 mins post contrast

2) 30-40 mins post contrast

3) 40-45 mins post contrast

Each group had 12 patients. Contrast to Noise Ratio (CNR) was computed as the ratio of the difference in signal intensity over a chosen region of interest in the scar and normal myocardium and the standard deviation of the noise observed in the blood pool. CNR was computed between scar and normal myocardium CNR_SM_ & between scar and blood CRN_SB_.

## Results

**Figure 1:**
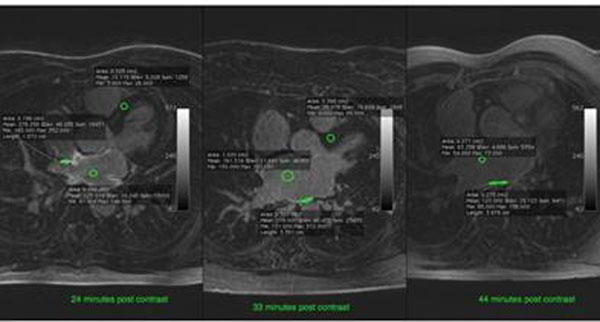
The measurement of CNR in representative images from the studied groups.

Table 1 shows the results of the analysis. Figure 1 shows a comparative image of the CNR measurement made in 3 datasets, one from each group. Unpaired Student’s t-test was performed on these datasets and the p values were found to be - p=0.24 (for CNR_SB_ between <30min and 30-40min); p=0.69 (for CNR_SB_ between 30-40min and >40min); p=0.97 (for CNR_SM_ between <30min and 30-40min) and p=0.54 (for CNR_SM_ between 30-40min and >40min). Thus, the difference in CNR between scar and normal myocardium and scar and blood observed in all three groups is not statistically significant.

**Table 1:** CNR computed for each studied group

Time post contrast	CNR_SM_ (mean ± std)	CRN_SB_ (mean ± std)
25.2 ± 4 minutes	20.3 ± 9.1	8.7 ± 4.8
34.3 ± 2.5 minutes	20.2 ± 8.8	11.3 ± 5.9
42. ± 2.8 minutes	17.3 ± 3.5	10 ± 1.9

## Conclusion

From these preliminary results, it follows that LGE imaging performed between 25 to 45 minutes post contrast injection give comparable visibility of post ablation scar.
